# Combination of transcriptomic, biochemical, and physiological analyses reveals sugar metabolism in *Camellia drupifera* fruit at different developmental stages

**DOI:** 10.3389/fpls.2024.1424284

**Published:** 2024-08-13

**Authors:** Zhen Liu, Chunhui Shen, Ruifan Chen, Zhiqiang Fu, Xiaomei Deng, Ruchun Xi

**Affiliations:** ^1^ Guangdong Key Laboratory for Innovative Development and Utilization of Forest Plant Germplasm, Guangzhou, China; ^2^ College of Forestry and Landscape Architecture, South China Agricultural University, Guangzhou, China

**Keywords:** sucrose transport, fruit growth, sugar content, RNA-seq, enzymes in sucrose metabolism

## Abstract

*Camellia drupifera*, a significant woody oil crop in southern China, produces oil from its fruit seeds. Understanding sugar metabolism enzyme regulation is crucial for sugar accumulation and oil synthesis in fruit organs. This study examines the dynamic changes in sugar metabolism across four developmental stages of *C. drupifera* fruits, from rapid fruit enlargement to oil conversion. We analyzed sugar content, enzyme activity, and transcriptomic data to identify key periods and mechanisms involved in sugar metabolism. Our findings indicate that photosynthetic products are rapidly transported from leaves to fruit organs after synthesis, with transport efficiency decreasing significantly after 48 hours. September was identified as a critical period for oil conversion, during which the highest sucrose levels and SuSy-II enzyme activity were detected in the kernels. A positive correlation was found between high expression of ten genes related to sugar metabolism enzymes and sugar transport proteins and sucrose content. Notably, the expression levels of c158337.graph_c0 (SPS), c166323.graph_c0 (SuSy), c159295.graph_c0 (SUC2-like), and c156402.graph_c0 (SUC2-like) significantly increased during the oil conversion phase.These findings provide a crucial theoretical foundation for elucidating the molecular mechanisms of sugar metabolism in *C. drupifera* fruits, offering insights that could enhance its economic yield.

## 1 Introduction

The growth and development of higher plants rely on carbohydrates produced through photosynthesis, primarily stored as sugars in their sink organs such as fruits, seeds, and flowers (Lu et al., 2022). Sugar metabolism in plants is regulated by various enzymes including sucrose synthase (SuSy), sucrose phosphate synthase (SPS), and invertase (INV), which play crucial roles in the synthesis and breakdown of sugars (Wang H. et al., 2018; Umer et al., 2020; Zhou et al., 2022). The diversity of sugar types varies significantly among different species, within different cultivars of the same species, and among different organs of the same plant (Hussain et al., 2020). In plant systems, the transport and distribution of photosynthetic products is a complex process involving the supply capacity of source organs, the transport efficiency of the phloem, and the competitive abilities of sink organs (Julius et al., 2017). Research on *Camellia oleifera* has highlighted correlations between fruit oil content and sucrose/starch content, as well as activities of sugar metabolism enzymes such as soluble acid invertase (S-AI), cell wall invertase (CWI), and neutral invertase (NI) (Aguirre et al., 2018; Robert, 2019). These findings underscore the critical role of photosynthate allocation and sugar metabolism in the growth and development of *C. oleifera* fruits. Sucrose, as a non-reducing sugar with high solubility and low viscosity, serves as a crucial hub in various metabolic pathways within the phloem (Wang X. et al., 2018; Song et al., 2021). The phloem predominantly facilitates the transport of sucrose, contributing significantly to the increased growth rates and biomass accumulation in plants (Murcia et al., 2016; Pan et al., 2021). Intercellular transport of sugars relies on specific sugar transport proteins such as Monosaccharide Transporters (MST), Sucrose Transporters (SUT) and Sugars Will Eventually be Exported Transporters (SWEET). MST proteins are responsible for the transmembrane transport of monosaccharides such as glucose and fructose. SUT proteins specifically transport sucrose. SWEET proteins can transport multiple types of sugars, including hexoses (such as glucose) and sucrose (Hennion et al., 2019; Li J. et al., 2022). These sugar transport proteins are closely associated with sugar accumulation and plant growth, CoSWEET10, SUTs, and SWEET-like have been shown to play roles in sugar transport and the regulation of plant growth in the source and sink organs of *C. oleifera* (He et al., 2022; Ye et al., 2023).

*Camellia drupifera* belongs to the Theaceae family and the Camellia L. genus and is a significant woody oil crop in southern China. Its flowering period typically spans from early November to late January of the following year. The fruit development process can be divided into distinct stages: a slow growth phase from flowering and pollination to mid-to-late July, followed by a rapid growth phase in August, and an oil conversion phase from mid-August to November. The seeds of *C. drupifera* are particularly rich in unsaturated fatty acids, known for their antioxidant, anticancer, and immune-boosting properties. This makes the oil highly valuable in pharmaceuticals, health foods, and daily chemical products, with substantial potential for preventing and treating cardiovascular diseases (Li Y. et al., 2022). Despite its economic promise, *C. drupifera* faces challenges such as low yield per unit area and alternate bearing phenomena, limiting its industrial development. Therefore, a comprehensive understanding of photosynthate allocation and sugar metabolism pathways in *C. drupifera* is crucial for enhancing its economic yield. Currently, systematic research in these areas is lacking, with most studies focusing on physiological aspects or analogous varieties like *Camellia oleifera* (He et al., 2022). However, *C. drupifera* exhibits distinct physiological characteristics, including phenology and fruit morphology, which necessitate targeted research efforts. During oil synthesis, sucrose synthesis plays a pivotal role. Hence, identifying key genes involved in sucrose transport and metabolism during fruit development is essential for comprehending and optimizing oil synthesis in *C. drupifera*. This overview highlights the botanical and economic significance of *C. drupifera*, underscoring critical areas requiring research focus to improve its productivity.

This study aims to investigate sugar accumulation and the activity of related enzymes in various organs of *C. drupifera* during fruit development. Improved sampling methods and optimized measurement parameters will be employed for accurate data collection. The study focuses on ten-year-old *C. drupifera* plants, examining changes in sugar content, activities of sugar metabolism-related enzymes (such as SuSy and SPS), ^13^C stable isotope abundance, and transcriptomic data. The research covers the period from the early rapid growth phase of fruit to the oil synthesis and pre-harvest phase, spanning the months from July to October. The primary objective is to identify candidate genes pivotal in sugar metabolism pathways specific to *C. drupifera*. These findings are expected to provide a robust theoretical foundation for enhancing the economic yield of this important woody oil crop in southern China.

## 2 Materials and methods

### 2.1 Plant materials and sample collection

#### 2.1.1 Experimental Site Conditions

The experimental materials were obtained from the South China Agricultural University, located in Zengcheng (23°14’48” N, 113°38’20” E). The site is an average elevation of 85 meters and is situated within a subtropical-tropical humid monsoon climate zone. The annual mean temperature is 21.9°C, and the average yearly rainfall is 2004.5 mm. The soil is classified as red soil with a pH of 4.6, an organic matter content of 8.57 g·kg^-1^, a field water-holding capacity of 20.41%, and soil bulk density of 1.55 g·cm^-3^.

#### 2.1.2 Overview of the Experimental Plantation

The experimental plantation covers 8 hectares and was established in April 2010 with one-year-old container seedlings of *C. drupifera*. Each planting pit is 50 cm × 50 cm × 40 cm in size and received 0.5 kg of organic fertilizer. The spacing between rows and plants is 3 meters by 3 meters. The current retention rate of the stand is 87.5%. The plantation is managed according to high-yield forest standards to ensure optimal growth and fruit development. The average ground diameter of the trees is 9.5 ± 1.2 cm, the average tree height is 3.0 ± 0.9 meters, and the average crown dimensions (east-west × north-south) are 2.3 ± 0.5 meters × 2.7 ± 0.3 meters. Prior to November 2020, silvicultural practices were implemented, including removing weak and shading branches, clearing undergrowth, and pruning trees.

#### 2.1.3 Sample Tree Selection

In November 2020, six sample trees were selected for analysis using an “S”-shaped random sampling method. This method was used to ensure the selected trees were representative of the population, with optimal sunlight exposure, uniform growth, similar fruit development, and no pests or diseases. Additionally, three trees located away from the sample trees were selected as controls. The average ground diameter of the sample trees was 10.2 cm, the average tree height was 3.2 meters, and the average crown dimensions (east-west × north-south) were 2.7 meters × 2.7 meters. These trees were subjected to standard management practices throughout the experimental period.

#### 2.1.4 Sample Collection

Samples were collected from trees located in the east, west, south, and north parts of the plantation between July and October 2021. Five fully functional leaves were randomly selected and wrapped in aluminum foil from each sampled tree. Additionally, five undamaged and well-developed fruits were collected from each tree, with the peels and seeds removed. Fruit peels and seeds were stored separately in 50 mL centrifuge tubes, flash-frozen in liquid nitrogen, and kept on dry ice before being transported to the laboratory. In the laboratory, leaf, fruit peels, and seed samples were ground into powder with liquid nitrogen and stored at -80°C for later analysis of sugar content and metabolic enzyme activity. After administering the ^13^C tracer (Section 2.2 for detailed methods), samples of leaves, branches, fruit peels, and seed kernels were collected at 0, 6-, 24-, 48-, and 72-hours after labeling in August. In July, August, September, and October, samples were collected 72 hours after labeling to measure ^13^C enrichment and total carbon content.

### 2.2 ^13^C Pulse-labeling and carbon abundance determination

**Figure 1:**
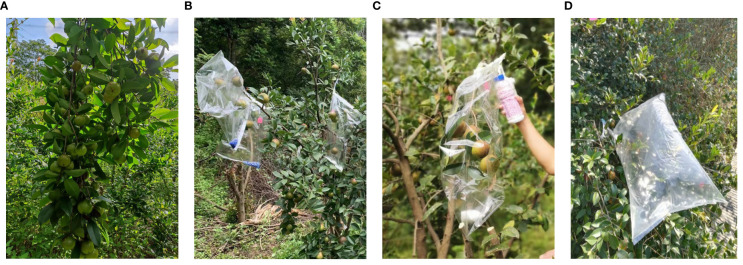
Illustrates the ^13^C pulse-labeling process, which comprises four main steps: **(A)** Pre-labeling Treatment; **(B)** ^13^C Labeling - Drying; **(C)** ^13^C Labeling - Vacuuming; **(D)** ^13^C Labeling - Feeding with ^13^C Gas.

The ^13^C labeling was conducted on clear mornings in July, August, September, and mid-October 2021 (**Figure 1**).

#### 2.2.1 Pre-labeling Treatment

Before labeling, the branches in the middle layer of the sample tree canopy were pruned to achieve a leaf-to-fruit ratio of 4:1. The branches were cut 5 cm away from the fruit.

#### 2.2.2 ^13^C Labeling - Drying

Polyethylene bags sprayed with an anti-fogging agent and dried were wrapped around the branches. Rubber hoses were used to connect the inside and outside of the bags. The bags were sealed both inside and outside with double-sided tape and wide adhesive tape. Inside the bag, CaCl_2_ and color-changing silica gel were placed to absorb water vapor produced by plant transpiration.

#### 2.2.3 ^13^C Labeling - Vacuuming

An air pump extracted the gas inside the bag. Air devoid of CO_2_ and H_2_O was then injected into the bag until it was fully inflated before sealing.

#### 2.2.4 ^13^C Labeling - Feeding with ^13^C Gas

13 ml of CO_2_ gas with a ^13^C abundance of 99% (purchased from the Shanghai Stable Isotope Engineering and Technical Research Center) was injected into the bag every hour for 2 hours.

#### 2.2.5 Carbon Content Determination

In August, samples were taken from leaves, branches, fruit peels, and seeds at 0, 6, 24, 48, and 72 hours after labeling. Sampling was conducted at 72 h after labeling in July, August, September, and October. The harvested samples were placed in an oven at 105°C for 30 minutes to halt metabolic activity and then dried to a constant weight at 75°C. After determining the dry weight, the samples were ground through a 100-mesh sieve and stored in sealed containers. Measurements were conducted once all samples for analysis were collected.

### 2.3 Sugar content determination

Sugar content and enzyme activity in the samples were determined using reagent kits from Suzhou Keming Biotechnology Co., Ltd., following the microplate method. Each measurement was performed in quadruplicate. The main instruments used during the analysis included a cryogenic grinder, an Eppendorf tabletop centrifuge, and a Multiskan FC Microplate Reader (Thermo Fisher Scientific, Waltham, MA, USA).

#### 2.3.1 Soluble sugar content determination

##### 2.3.1.1 Sample Treatment

Weigh 0.1 g of powdered sample into a 1.5 ml EP tube, add 1 ml of deionized water, and incubate in a 95°C water bath for 10 minutes. After cooling, centrifuge at 8000 rpm for 10 minutes. Transfer the supernatant to a 10 ml tube, dilute to 10 ml with distilled water, mix well, and prepare for measurement.

##### 2.3.1.2 Soluble Sugar Content Determination

The anthrone colorimetric method was used. First, add 40 μL of the sample to an EP tube, then add 40 μL of distilled water, 20 μL of the working solution, and 200 μL of concentrated sulfuric acid in sequence. Heat the mixture in a 95°C water bath for 10 minutes, then cool to room temperature. Extract 200 μL of the solution and measure the absorbance (A) at 620 nm.

Soluble sugar content (mg·g-1 fresh weight) was calculated using the formula: 2.34 × (*A* + 0.07)/*W*, where W is the fresh weight of the sample (g).

#### 2.3.2 Sugar component determination

##### 2.3.2.1 Sample Treatment

Weigh 1 g of the sample into a 15 ml EP tube and add 5 ml of double-distilled water. Mix well and incubate in an 80°C water bath for 20 minutes, mixing gently every 5 minutes. After cooling, centrifuge at 5000 rpm for 15 minutes. Transfer the supernatant to a new EP tube, add 4 ml of double-distilled water to the residue, and repeat the extraction process. Combine the supernatants and dilute to 10 ml. Take 2 ml of the diluted solution and centrifuge at 12000 rpm for 20 minutes. Using a 1 ml syringe, extract approximately 200 μL of the solution and purify it through a 0.22 μL water film filter. Transfer the purified solution to a sample bottle for measurement.

##### 2.3.2.2 Sugar Component Determination

Prepare standard solutions of sucrose, glucose, and fructose at concentration gradients of 0.625 mg·ml-1, 1.25 mg·ml-1, 2.5 mg·ml-1, 5 mg·ml-1, 10 mg·ml-1, and 20 mg·ml-1. Use high-performance liquid chromatography (HPLC) for the analysis. Chromatographic conditions are as follows: ultrapure water as the mobile phase, a flow rate of 0.3 ml·min-1, isocratic elution, a column temperature of 80°C, an injection volume of 10 μL, and a testing time of 20 minutes. Perform qualitative analysis and standard curve plotting using standard samples. Calculate the peak area of each test sample and convert it into milligrams of soluble sugar content per gram of fresh weight (mg·g^-1^ fresh weight).

#### 2.3.3 Starch content determination

##### 2.3.3.1 Sample Treatment

Weigh 0.1 g of powdered sample into a 2 ml EP tube and add 1 ml of Reagent 1. Mix well and incubate in an 80°C water bath for 30 minutes, then centrifuge at 3000 rpm for 5 minutes at 25°C. Discard the supernatant and add 0.5 ml of distilled water to the precipitate. Gelatinize the precipitate in a 95°C water bath for 15 minutes. After cooling, add 0.35 ml of Reagent 2 and extract at room temperature for 15 minutes. Add 0.85 ml of distilled water and centrifuge at 3000 rpm for 10 minutes at 25°C. Mix 50 μL of the supernatant with 250 μL of the working solution, heat in a 95°C water bath for 10 minutes, then cool to room temperature. Measure the absorbance (A) at 620 nm using 200 μL of the solution.

##### 2.3.3.2 Starch Content Determination

Starch content (mg·g^-1^ fresh weight) was calculated using the formula: 0.578 × (*A* + 0.0295)/*W*.

### 2.4 Sucrose metabolism

#### 2.4.1 Sample Processing

The organs of *C. drupifera* stored at -80°C were taken out and separately ground in liquid nitrogen. Weigh 0.1 g of the ground sample. Enzyme activity was determined using a tissue mass to extraction solution volume ratio of 1:5 to 1:10.

#### 2.4.2 Enzyme Activity Determination

Enzyme activities were measured using assay kits for SuSy-II (SSII-2-Y), SPS (SPS-2-Y), NI (NI-2-Y), B-AI (BAI-2-Y), and S-AI (SAI-2-Y).

### 2.5 Library construction, sequencing, and data processing

Transcriptome RNA libraries were constructed and sequenced from triplicate fruit samples collected on sunny days in July, August, September, and October of 2022. RNA extraction was conducted using the Omega Bio-Tek Co. plant RNA isolation kit (Norcross, GA, USA). The NanoPhotometer N60 (Implen, Munich, Germany) was used to assess RNA purity, concentration, and integrity, ensuring sample suitability for transcriptome sequencing. Library construction and RNA-seq were performed by Biomarker Technologies Co., Ltd. Sequencing libraries were generated using the NEBNext^®^Ultra™ RNA Library Prep Kit for Illumina^®^ (NEB, Ipswich, MA, USA) according to the manufacturer’s protocol, with index codes added to distinguish sequences for each sample. mRNA was purified from total RNA using poly-T oligo-attached magnetic beads. Fragmentation was conducted using divalent cations under elevated temperature in NEBNext First Strand Synthesis reaction buffer (5X). First-strand cDNA was synthesized using random hexamer primers and M-MuLV Reverse Transcriptase (Thermo Fisher Scientific, Waltham, MA, USA). Second-strand cDNA synthesis was subsequently performed using DNA Polymerase I and RNase H. The remaining overhangs were converted into blunt ends through exonuclease/polymerase activities. After adenylation of the 3’ ends of DNA fragments, NEBNext Adaptor with a hairpin loop structure was ligated to facilitate hybridization. The library fragments were purified using the AMPure XP system (Beckman Coulter, Beverly, MA, USA) to select cDNA fragments of 240 bp in length. Three microliters of USER enzyme (NEB, USA) were added to size-selected, adapter-ligated cDNA and incubated at 37°C for 15 min, followed by 5 min at 95 °C. Subsequently, PCR amplification was conducted using Phusion High-Fidelity DNA polymerase (Thermo Fisher Scientific, Waltham, MA, USA), universal PCR primers, and Index (X) Primer. PCR products were purified using the AMPure XP system, and the library quality was evaluated using an Agilent 2100 Bioanalyzer system. Index-coded samples were clustered on an Illumina cBot cluster generation system using the TruSeq PE cluster kit v.4-cBot-HS (Illumina), following the manufacturer’s protocol. The libraries were sequenced on an Illumina platform, producing paired end reads. Adaptor sequences and low-quality reads were eliminated from the datasets following quality control procedures. Raw sequences were processed to generate clean reads. Gene function was annotated using databases such as NR (NCBI non-redundant protein sequences), NT (NCBI nonredundant nucleotide sequences), Pfam (Protein family), KOG/COG (Clusters of Orthologous Groups of proteins), Swiss-Prot (a manually annotated and reviewed protein sequence database), KO (KEGG Orthologue database), and GO (Gene Ontology) resources. Differential expression analysis was conducted using BMKCloud with DESeq2 and EBSeq software, with parameters set to a false discovery rate (FDR) of ≤ 0.05 and a log^2^ fold change (FC) of ≥ 1.

### 2.6 Statistical analysis

#### 2.6.1 Calculation of ^13^C allocation in various organs

Calculation of natural abundance *δ* ^13^C(‰):


$${\text{δ}}^{13}C=\frac{R_{S}-R_{c}}{R_{c}}\times 1000‰$$

Where “*R_s_*” represents the ratio of ^13^C/^12^C; “*R_c_*” is the standard carbon isotope ratio of 0.0112372.

^13^C atomic percentage Atom%^13^C (%):


$${\text{Atom\%}}^{13}\text{C}=\frac{[\left({\text{δ}}^{13}\text{C}+1\;000)\times R_{c}\right]}{\left({\text{δ}}^{13}\text{C}+1\;000\times R_{c}\right)+1\;000}\;100\%$$

Total fixed carbon content C_i_ in each organ: C_i_ (mg):


$${\text{C}}_{\text{i}\;=}\text{C}\%\times {\text{W}}_{\text{i}}$$

Where “C%” represents the carbon percentage in each organ; “*W_i_*” is the biomass of each organ; “*i*” denotes different organs.

Fixed ^13^C_i_ (mg) in each organ: ^13^C_i_ (mg):


Ci13=Ci×(Atom%13C−Fn)100×1000

Where “*F_n_*” represents the ^13^C atomic percentage in unlabeled samples.

Allocation ratio P_i_ (%) of ^13^C in each organ for each month: P_i_ (%):


Pi=Ci13Cf13

Where “*^13^C_f_* ” represents the sum of accumulated ^13^C in each location.

Transport rate of ^13^C photosynthetic products to various organs: V_i_ (ug·g^-1^·h^-1^) = Ci13H

Where “*^13^C_i_*%” represents the ^13^C content in each organ, and “*H*” is the time after labeling ends.

#### 2.6.2 Statistical analyses

The experimental results are presented as mean ± standard error of the mean (SEM). All data were tested for normality using the Shapiro-Wilk test in SPSS software (version 25.0). For normally distributed data, a one-way analysis of variance (ANOVA) was performed. This was followed by multiple comparisons using the least significant difference (LSD) method to determine significant differences between group means Graphs and charts were generated using Origin 2021 software to visualize the experimental results and analytical conclusions. Furthermore, transcriptome sequencing, quality assessment, transcriptome assembly, functional annotation of Unigenes, and differential gene expression analysis were conducted using a no-reference genome transcriptome analysis platform provided by Beijing Biomarker Technologies Co., Ltd.

## 3 Result

### 3.1 Variations in *δ* ^13^C isotope in different organs of *C. drupifera* at various developmental stages

**Figure 2:**
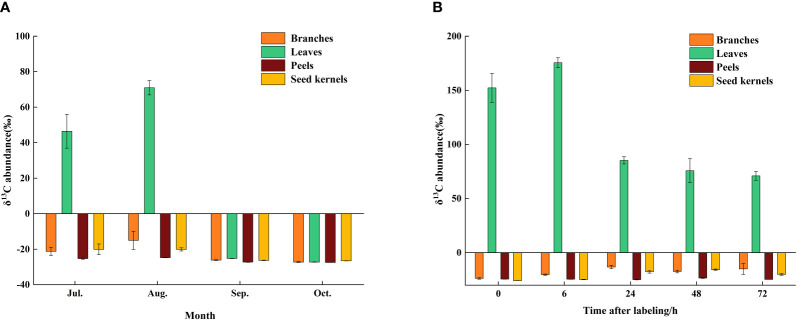
Accumulation of of *δ* ^13^C in various organs during different growth periods of *C. drupifera*. **(A)** Samples were collected and the abundance of *δ* ^13^C in *C. drupifera* branches, leaves, seed kernels, and fruits was assessed at 0, 6, 24, 48, and 72 hours following the ^13^C labeling conducted in August. The Y-axis indicates the abundance of *δ* ^13^C, while the X-axis represents different sampling times. **(B)** The *δ* ^13^C abundance in the branches, leaves, seed kernels, and fruits of *C. drupifera* was determined 72 hours after ^13^C labeling at various growth and development stages (July, August, September, and October). The data are shown as the means ± standard deviations (SDs).

To investigate the transport and accumulation of photosynthetic products between source and sink organs in *C. drupifera* during different developmental stages, the research team employed detailed analysis with ^13^C pulse labeling technology. At the end of the ^13^C labeling (0 h), the δ^13^C content in the leaves measured 152.26‰. Within the subsequent 6 hours, the δ^13^C content peaked at 175.50‰, demonstrating rapid fixation and accumulation in the leaves. This suggests that synthesis of photosynthetic products was primarily concentrated within the initial 6 hours after labeling. Between 0 and 6 hours after labeling, photosynthetic products were transported from the source organ (leaves) to the sink organs (branches and seeds). Specifically, the δ^13^C content in the branches and seeds increased from -23.4‰ and -25.97‰ to -13.25‰ and -17.76‰, respectively, whereas the change in δ^13^C content in the fruit peels was minimal. From 6 to 24 hours, the δ^13^C content in the leaves sharply decreased by 90.23%, indicating significant transfer of photosynthetic products to other organs during this period. Between 24 and 72 hours, the δ^13^C content in the leaves stabilized, indicating completion of the transfer of fixed δ13from the leaves to sink organs (such as branches and seeds) (**Figure 2A**).

**Figure 3:**
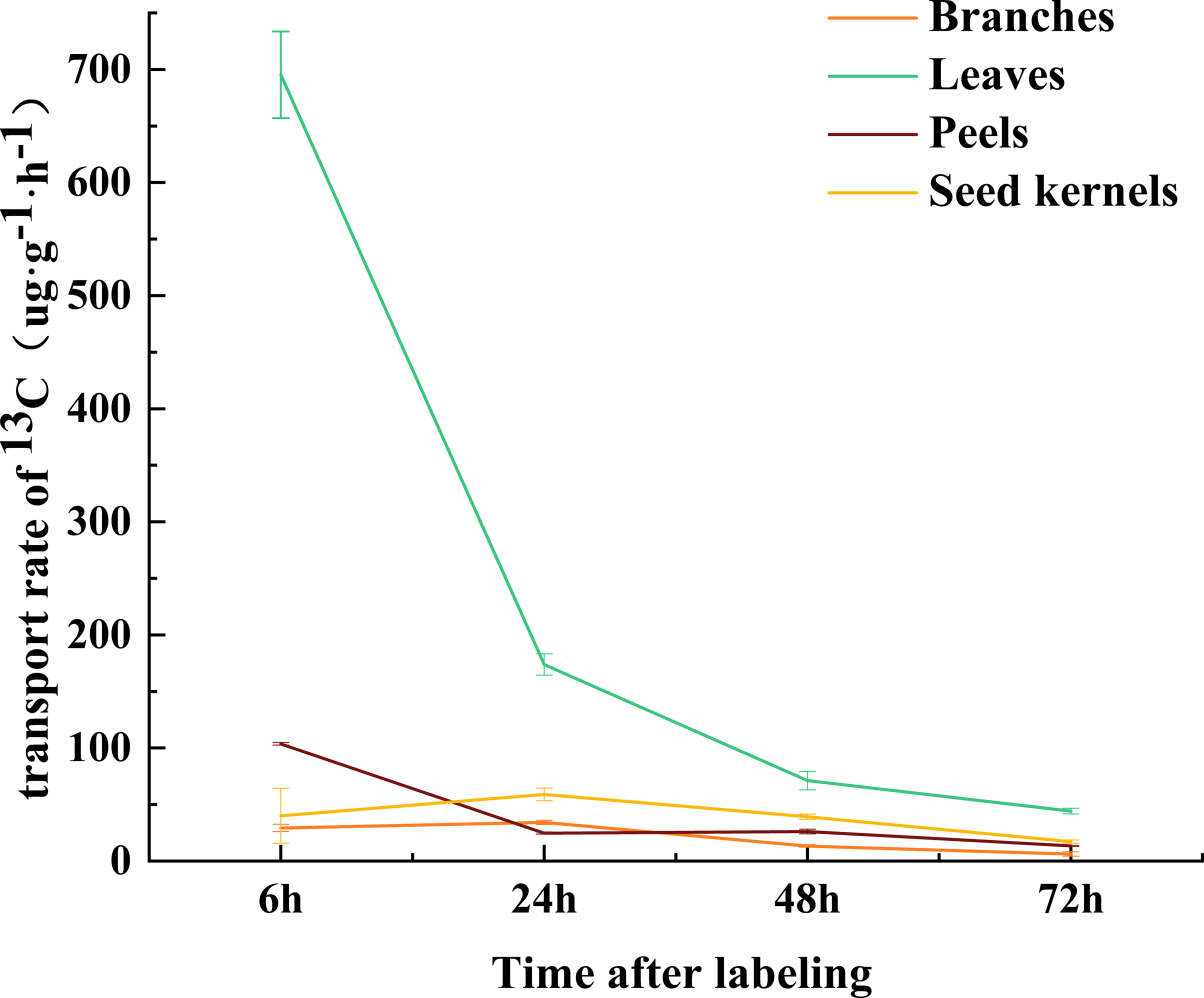
Transport rates of *C. drupifera* over time following ^13^C labeling. The abscissa represents different time intervals (6-72h) after the end of ^13^C labeling, and the ordinate represents the *δ* ^13^C transport rate.

The transport rate of δ^13^C in the leaves was highest during the initial 6 hours and gradually slowed down thereafter, reaching its lowest point after 72 hours (**Figure 3**). This trend suggests that rapid transport of photosynthetic products primarily occurs within the first 6 hours after carbon fixation in the source organs, with the transport rate progressively decreasing over time. At different developmental stages, the δ^13^C content in the source and sink organs exhibited significant variations. Overall, the δ^13^C content in various organs increased gradually in July and August, reaching its peak in August. Subsequently, the δ^13^C content in all organs decreased rapidly, with significant fluctuations observed in the leaves, ranging from a high of 70.98‰ in August to a low of -27.29‰ in October (**Figure 2B**). Photosynthetic products are transported through the phloem from the source (leaves) to the sink organs, where they are stored. At different developmental stages, significant differences were observed in the distribution proportions of photosynthetic products among the organs of *C. drupifera* (**Table 1**). During the early fruit development stage (July), the proportion of photosynthetic products allocated to the leaves was significantly higher than in other organs. However, as development progressed, this proportion dropped sharply, reaching only 3.84% by October. In September, the fruit peels became a major storage sink for photosynthetic products, with their allocation peaking at 42.56%. The accumulation of photosynthetic products in the branches remained consistently low. As the fruit developed, the seeds emerged as the primary storage center for photosynthetic products during the oil conversion period in October, accumulating 64.43% of the photosynthetic products.

**Table 1:** The accumulation of ^13^C mass in *C. drupifera* after labeling and the percentage of ^13^C in each organ.

Month	Total accumulation of ^13^C mass in plants (mg·plant^-1^)	The percentage of ^13^C in each organ (%)			
		Branches	Leaves	Peels	Seed kernels
7	3.42±0.30	7.77±2.74Cb	69.84±2.00Aa	15.94±0.39Bc	6.45±2.50Cc
8	5.84±0.22	7.70±2.58Db	54.55±1.80Ab	16.54±0.64Cc	21.21±1.42Bb
9	0.38±0.26	13.17±2.21Ba	21.48±1.25Bc	42.55±7.85Aa	22.81±7.87Bb
10	0.17±0.13	4.90±1.33Cb	3.78±2.47Cd	26.86±3.83Bb	64.47±6.63Aa

Different capital letters in the same row indicate a significant difference between different organ (*p*<0.05), while different lowercase letters in the same column represent a significant difference between sampling times (*p*<0.05).

### 3.2 Sugar content in various organs of *C. drupifera* at different developmental stages

**Figure 4:**
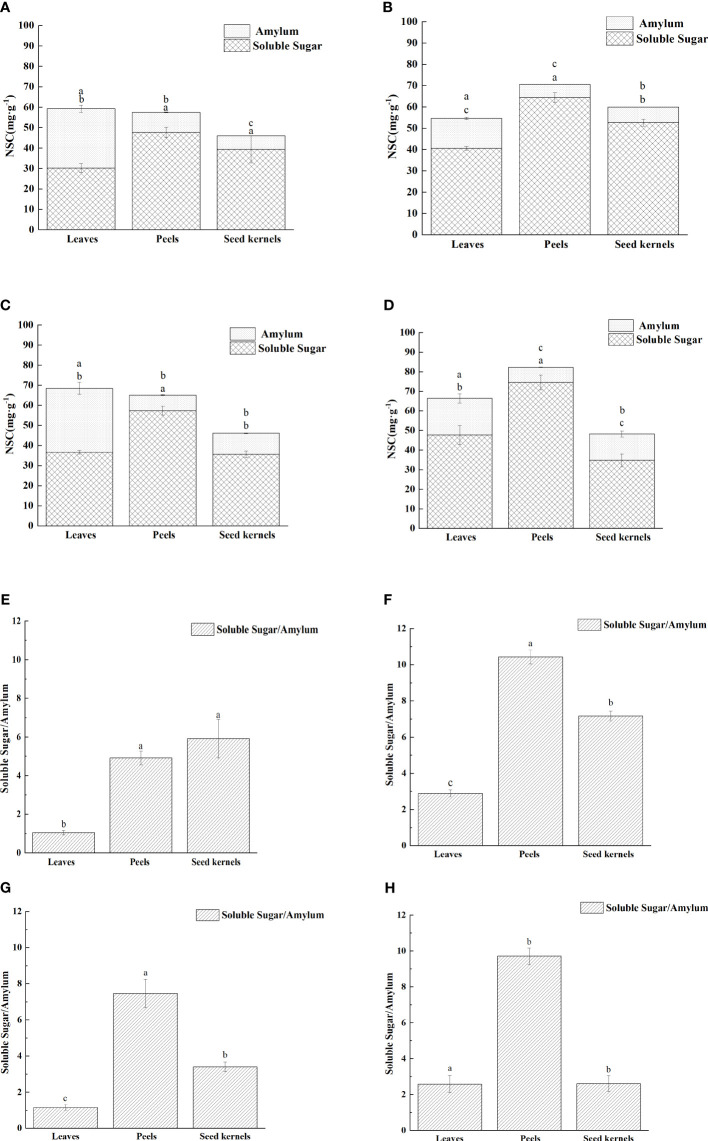
Distribution of non-structural carbohydrates in various organs of *C. drupifera* at different growth stages. The NSC (soluble sugar and starch) contents of each part of *C. drupifera* during July, August, September, and October are shown panels **(A–D)**, respectively. Different lowercase letters from top to bottom indicate significant differences in starch and soluble sugar contents among different organs (*p*<0.05). Panels **(E–H)** display the soluble sugar to starch ratio of each part of *C. drupifera* during July, August, September, and October, respectively. Lowercase letters indicate significant differences in the ratio of soluble sugar to starch among different organs (*p*<0.05).

An analysis of non-structural carbohydrates (NSC, including soluble sugars and starch) in various organs of *C. drupifera* at different developmental stages revealed significant differences (P< 0.05) across these stages (**Figures 4A–E**). The proportion of soluble sugars in the total NSC content of each organ exceeded 50% (**Figure 4B**), indicating that soluble sugars are the primary form of NSC in *C. drupifera*. In July, differences in NSC content among the organs were the smallest (**Figure 4A**).

**Figure 5:**
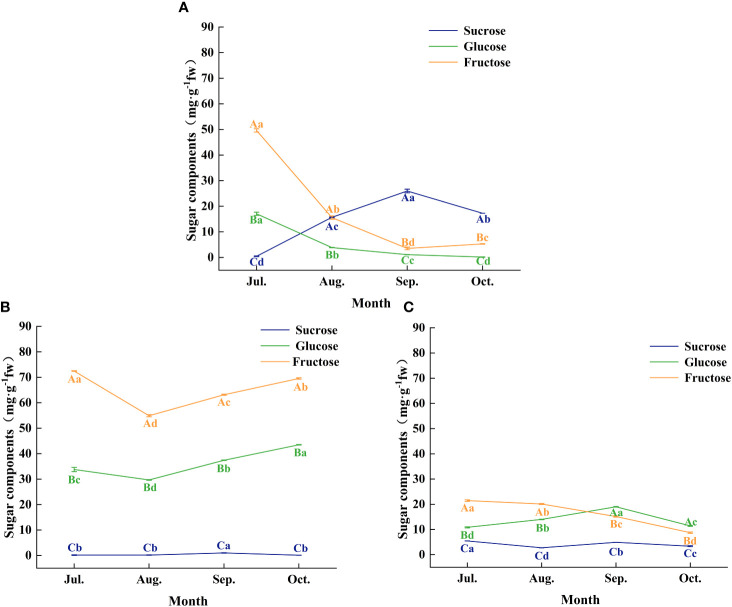
The characteristics of sugar components of each organ of *C. drupifera* at different growth stages. Panels **(A–C)** show the sugar fractions of the seed kernel, leaf, and peel of *C. drupifera* respectively. The sucrose, glucose, and fructose are represented by the three-color curves. Different capital letters in the data correspond to significant differences between different sugar fractions of the same organ in the same sampling period (*p<* 0.05). Significant differences between the same organ and sugar fractions of different sampling periods were indicated by different lowercase letters (*p*< 0.05).

Further analysis of changes in the content of the three major soluble sugars (sucrose, glucose, and fructose) in leaves, fruit peels, and seeds revealed significant differences in sugar accumulation among these organs at different developmental stages (**Figures 5A–C**). During July and August, fructose content was highest in leaves, fruit peels, and seeds, suggesting that during the rapid growth phase of *C. drupifera* fruits, photosynthetic products primarily accumulate as fructose. As the fruit developed, particularly in September, sucrose content in the seeds gradually increased, reaching a peak value of 25.93 mg·g^-1^ fresh weight (**Figure 5A**). This indicates that during the transition from the fruit growth period to the oil conversion period, the primary soluble sugar accumulated in seeds shifts from fructose to sucrose.

### 3.3 Activity of sugar metabolism-related enzymes in different organs of *C. drupifera* at various developmental stages

**Figure 6:**
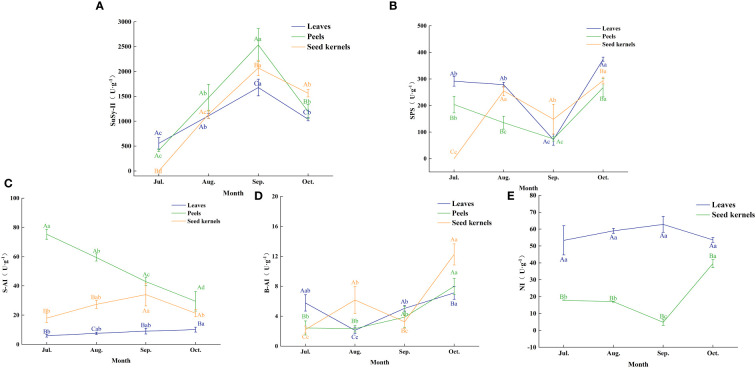
Enzyme activities related to sugar metabolism in various organs of *C. drupifera*. Panels **(A–E)** show the activities of SuSy-II, SPS, S-AI, B-AI, and NI enzymes in each organ of *C. drupifera*. The three-color curves indicate the peels, leaves, and seed kernels, respectively. Capital letters in the data indicate significant differences between the activities of the same enzymes in different organs at the same sampling period (*p*< 0.05). Lowercase letters in the figure indicate significant differences between the activities of the same enzymes in the same organ at different sampling times (*p*< 0.05).

The activity of enzymes involved in sugar metabolism plays a crucial role in the accumulation and distribution of sugars in fruit. By analyzing the changes in the activity of key sugar metabolism enzymes in various organs of **Figure 6**, we explored the correlation between these enzyme activities and sugar accumulation (**Table 2**).

**Table 2:** Correlation analysis of sugar content in various organs and enzyme activity in sugar metabolism.

organs	sugar components	SuSy-II	SPS	S-AI	B-AI	NI
Leaves	soluble sugars	0.482	0.052	0.514	-0.146	0.226
	fructose	-0.364	-0.182	-0.700*	-0.55	0.038
	glucose	0.895**	-0.926**	0.245	-0.284	0.590*
	sucrose	-0.133	-0.435	-0.306	0.447	-0.009
Peels	soluble sugars	0.171	0.14	-0.608*	0.437	
	fructose	-0.554	0.585*	0.071	0.341	
	glucose	0.005	0.477	-0.757	0.857**	
	sucrose	0.812**	-0.749**	-0.248	-0.109	
Seed kernels	soluble sugars	0.066	0.077	0.012	-0.209	-0.4
	fructose	-0.835**	-0.786**	-0.506	-0.577	-0.142
	glucose	-0.787**	-0.835**	-0.457	-0.615*	-0.209
	sucrose	0.957**	0.601	0.677*	0.288	-0.149

* Represents p< 0.05 and ** represents p< 0.01.

Sucrose synthase SuSy (in the synthesis direction, SuSy-II) exhibited a trend of rising and then falling activity in leaves, fruit peels, and seeds, reaching peak levels in September. The peak activities for each organ were 1,677.62 U·g^-1^ in leaves, 2,535.58 U·g^-1^ in fruit peels, and 2,071.62 U·g^-1^ in seeds, respectively. During the early fruit development phase (July to September), SuSy-II activity in leaves exceeded that in fruit peels and seeds. September marked a critical turning point in SuSy-II activity changes. After this period, the activity of SuSy-II in fruit peels and seeds surpassed that in leaves. In particular, the activity in seeds significantly exceeded that in other organs in October, indicating that during the fruit maturation stage, sugars predominantly accumulate in the seeds.

Sucrose phosphate synthase (SPS) in leaves and fruit peels showed a trend of decreasing and then increasing activity, with the lowest levels recorded in September at 71.44 U·g^-1^ and 74.43 U·g^-1^, respectively. In October, SPS activity significantly increased in both leaves and fruit peels. In seeds, SPS activity displayed a fluctuating upward trend, peaking in both August and October, with the latter peak slightly higher at 263.96 U·g^-1^. This suggests an increased demand for sucrose synthesis during the oil conversion phase of fruit development.

Soluble acid invertase (S-AI) in leaves gradually increased but maintained overall low activity with minimal variation. In fruit peels, S-AI activity gradually declined, reaching 29.48 U·g^-1^ in October, a 60.81% decrease from July. In seeds, S-AI activity showed an initial increase followed by a decrease, peaking at 34.06 U·g^-1^ in September.

Cell-wall Binding Acid Invertase (B-AI) remained generally low but varied across different organs. In leaves, B-AI activity first decreased and then increased, hitting a trough of 2.11 U·g^-1^ in August. In fruit peels, B-AI activity gradually rose, peaking at 7.99 U·g^-1^ in October. In seeds, B-AI activity exhibited a fluctuating upward trend, with peaks in August and October, the latter being higher at 12.25 U·g^-1^. When S-AI activity is low, the role of neutral invertase (NI) in sucrose breakdown becomes more prominent. In leaves, NI activity was about 5 to 10 times higher than that of S-AI, showing an upward trend that peaked at 62.80 U·g^-1^ in September, with slight variations between months. In seeds, NI activity first decreased and then increased, reaching a trough of 4.81 U·g^-1^ in September and peaking at 39.61 U·g^-1^ in October.

Correlation analysis between soluble sugar content and the activity of sugar metabolism-related enzymes in various organs of *C. drupifera* (**Table 2**) showed that SuSy-II, SPS, and S-AI activities were positively correlated with sucrose content in the seeds. Notably, SuSy-II activity exhibited a highly significant positive correlation with sucrose content in both seeds and fruit peels. SuSy-II activity remained consistently high throughout the development of *C. drupifera*, indicating its crucial role in sugar accumulation.

### 3.4 Transcriptome sequencing and quality assessment

To investigate the molecular mechanism of sugar metabolism and accumulation in *C. drupifera* fruits during their development, we utilized the Illumina NovaSeq high-throughput sequencing platform to sequence cDNA libraries from samples collected at different stages (July-October). This approach generated a total of 74.32 GB of raw data. Following the removal of adapter sequences and low-quality reads, each sample yielded 5.85 GB of clean data. The base quality (Q30) exceeded 94.45%, and we obtained a total of 55,787 unique sequence genes (unigenes) with an average length of 1060.6 bp. These metrics indicate the accuracy and high quality of the sequencing data, which are sufficient to support subsequent analyses.

### 3.5 Integrated analysis of gene expression and pathway enrichment in fruit development and sugar metabolism

**Figure 7:**
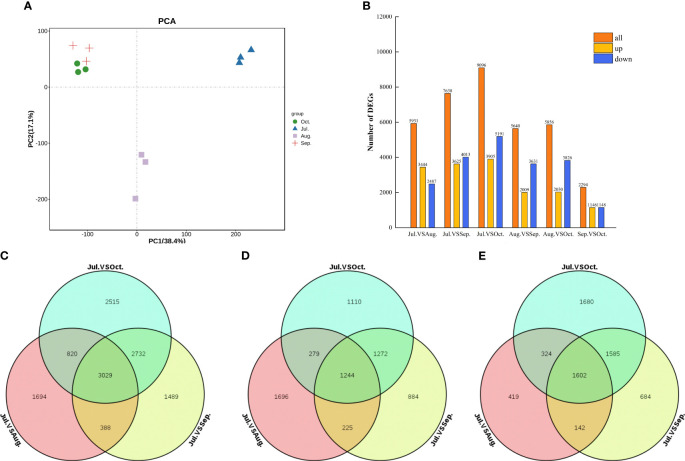
DEGs from transcriptome sequencing across four fruit developmental stages in *C. drupifera*. **(A)** Summary of DEGS in all combinations of developmental stage comparisons; **(B)** PCA from July to October. Samples from the same development stage were grouped together within the same circle; The Venn diagram illustrates the DEGs between Jul. vs Aug., Jul. vs Sep., and Jul. vs Oct. **(C)** All DEGs; **(D)** Up-regulated DEGs; **(E)** Down- regulated DEGs.

Using Principal Component Analysis (PCA), the transcripts from *C. drupifera* fruit were classified into four distinct groups. Each group consisted of three biological replicates, demonstrating significant clustering (**Figure 7A**). This clustering indicates strong correlations and distinctions among the samples. We further analyzed variations in gene expression across six different comparisons (Jul. vs. Aug., Jul. vs. Sep., Jul. vs. Oct., Aug. vs. Sep., Aug. vs. Oct., Sep. vs. Oct.) to understand the dynamics of sugar accumulation during different developmental stages of *C. drupifera* fruit. Differential gene expression analyses showed that DEGs were predominantly downregulated across all comparison groups, with notable prominence in the Jul. vs. Aug., Jul. vs. Sep., and Jul. vs. Oct. comparisons (**Figure 7B**). Venn diagrams illustrated that 3029 DEGs were commonly expressed during these three comparison periods **Figure 7**.

**Figure 8:**
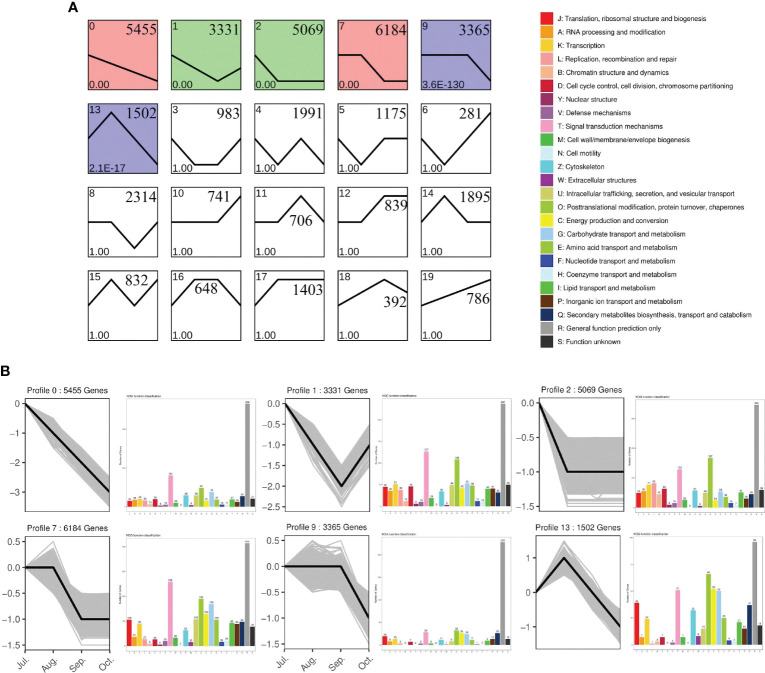
Clusters of total genes and EuKaryotic Orthologous Group (KOG) annotations of profiles. **(A)** All genes were clustered based on their FPKM data with an FDR ≤ 0.05. Profile numbers, p-values, and gene numbers are in the upper left corner, lower left corner, and upper right corner; **(B)** Six clusters were obtained by short time-series expression miner (STEM) software, analyzing gene expression profiles. The y-axis of each cluster indicates the log2FC value. KOG analysis utilized a p-value ≤ 0.05 and a minimum fold change of two. The y−axis of each KOG analysis represents the number of genes.

Based on the expression levels of all genes across the four experimental periods, their expression patterns were categorized into 20 distinct profiles (**Figure 8A**) Expression profiles 0, 1, 2, 7, 9, and 13 exhibited significant enrichment trends, particularly evident among genes annotated under KOG (Eukaryotic Orthologous Groups) classifications (**Figure 8B**). In these profiles, processes such as signaling pathways, transcription, translation, ribosomal structure, and morphogenesis were predominant during stages 0, 1, 2, 7, and 9, indicating a gradual reduction in cell division-related biological processes as Camellia oleifera fruits develop toward oil accumulation. Conversely, expression profile 13 showed significant increases in post-translational modification, protein turnover and chaperones, energy production and conversion, signaling pathways, carbohydrate transport and metabolism, as well as secondary metabolite biosynthesis, transport, and breakdown. These findings underscore the critical roles of carbohydrate and protein synthesis and transport during fruit oil accumulation.

**Figure 9:**
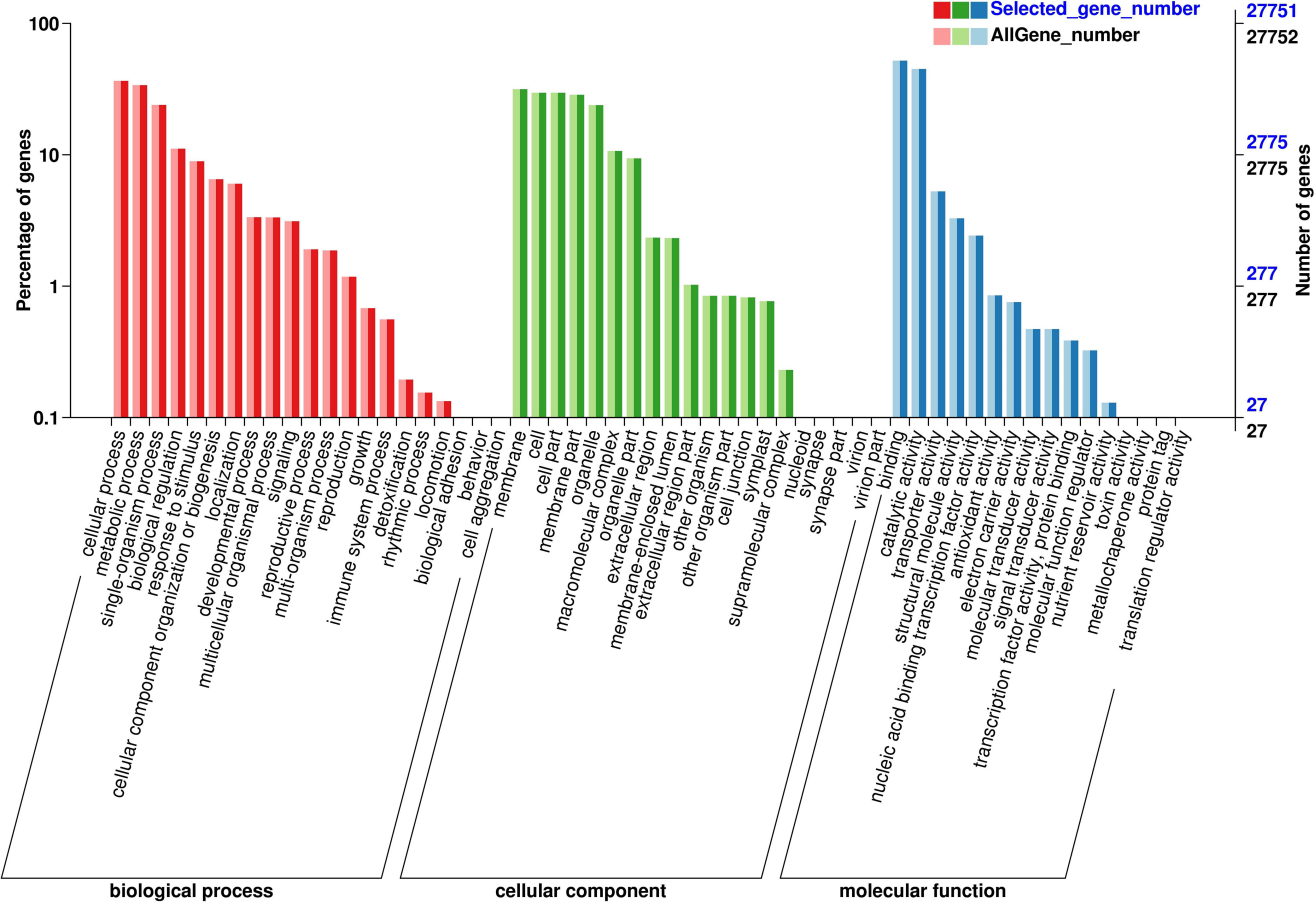
GO enrichment analysis of differential genes. The abscissa represents the ratio of the genes. The abscissa represents the ratio of the genes annotated to the entry to the total number of genes annotated, and the ordinate represents the name of the go entry.

**Figure 10:**
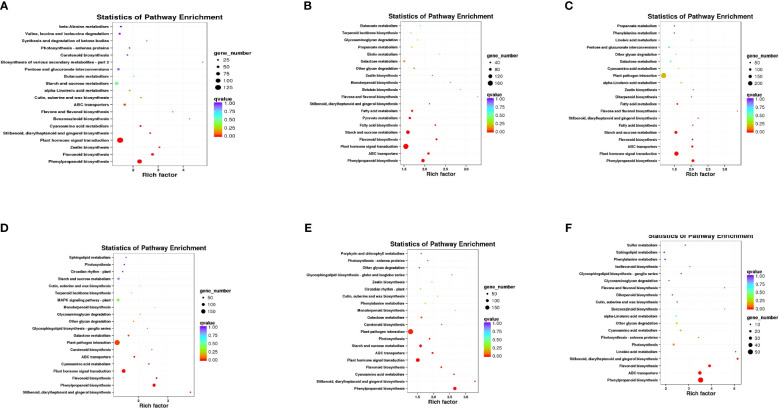
KEGG enrichment analysis of six developmental stage comparisons. **(A–F) **show the KEGG enrichment results of differential genes in the comparison groups (Jul.vsAug., Jul.vsSep., Jul.vsOct., Aug.vsSep., Aug.vsOct., and Sep.vsOct.). The ordinate represents the pathway names, while the abscissa represents the enrichment factor. The size of the dots indicates the number of differentially expressed genes (DEGs) in the pathway, and the color of the dots corresponds to different q-value ranges.

Further analysis was conducted on DEGs from six comparison groups using the GO and KEGG databases. Functional classification revealed by GO annotations primarily includes cellular components, biological processes, and molecular functions. Concerning cellular components, “cell,” “cell part,” and “membrane” were the three most significantly enriched categories, with the highest number of DEGs observed in the Jul.vsOct comparison group (**Figure 9**). In terms of biological processes, enrichment primarily occurred in cellular processes, metabolic processes, and single organism processes, while binding, catalytic activity, and transporter activity were predominant in molecular functions. After enriching all DEGs from the six comparison groups using KEGG, a total of 776 metabolic pathways were identified across the four growth and development stages of *C. drupifera* (**Figure 10**). DEGs were primarily enriched in pathways such as phenylpropane biosynthesis, plant hormone signal transduction, starch and sucrose metabolism, and ABC transporters. DEGs related to starch and sucrose metabolism were notably abundant in the Jul.vsSep. and Jul.vsOct. comparison groups, where the majority of these DEGs were identified.

**Figure 11:**
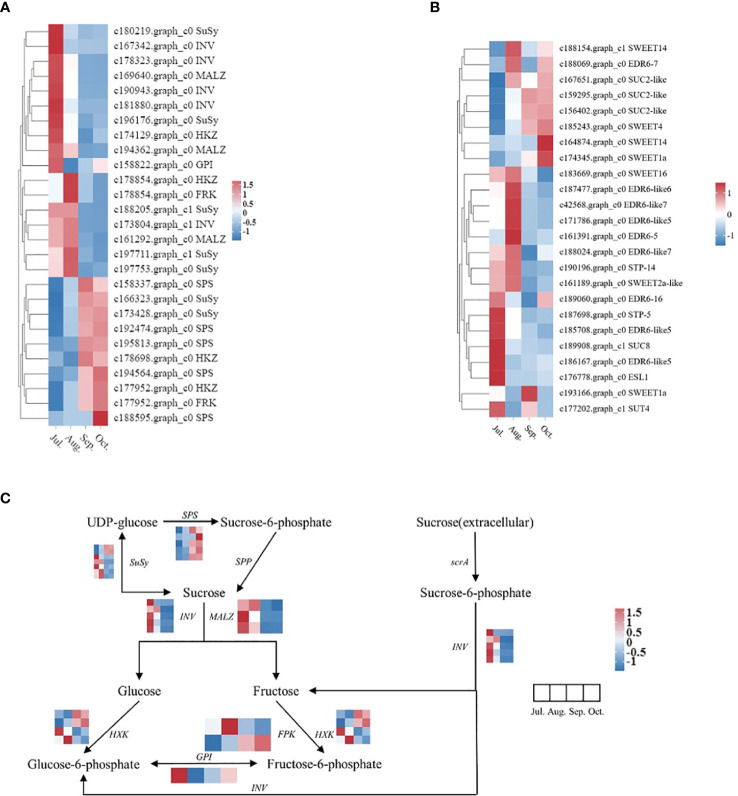
Sugar metabolism and transport protein genes, along with genes associated with sugar metabolism pathways, exhibited differential expression across various periods. The heat map was observed by TBtools based on RNA−seq FPKM. The right column shows the corresponding gene IDs and gene names, respectively. The color bar represents the expression level. **(A)** Heatmap related to sugar metabolism; **(B)** Heatmap related to sugar transport proteins; **(C)** A simplified sucrose biosynthesis and inversion pathway.

The biosynthesis and breakdown of sucrose are essential life processes in plants, involving key enzymes such as SPS, SuSy, INV, and the role of sugar transport proteins in sucrose transmembrane transport. Candidate genes related to sugar metabolism enzymes and sugar transport proteins were selected from all differentially expressed genes, followed by dynamic clustering heatmap analysis (**Figures 11A, B**). Based on their expression levels, DEGs were classified into two groups: Group I genes exhibit high expression in early developmental stages with decreased expression later, while Group II genes show the opposite pattern.

In Group I, 5 genes primarily encode the sucrose conversion enzyme INV, showing high expression in the early stages of fruit growth and development. Group II includes 2 genes encoding sucrose synthase SuSy, 5 genes encoding sucrose phosphate synthase SPS, and 8 genes encoding sugar transport proteins, with expression levels gradually increasing during the lipid conversion phase of fruit development. After correlating sucrose content and gene expression levels across different developmental stages of seeds, we identified genes whose expression levels showed strong positive correlations with changes in sucrose content (**Table 3**). Among genes related to sucrose synthesis and metabolism, the following genes exhibited the strongest positive correlations with sucrose content in seeds: c158337.graph_c0 (SPS), c166323.graph_c0 (SuSy), c193943.graph_c0 (SPS), c173428.graph_c0 (SuSy), c192474.graph_c0 (SPS). Among sugar transport protein genes, the following genes exhibited the most significant correlations with changes in sucrose content in seeds: c159295.graph_c0 (SUC2-like), c156402.graph_c0 (SUC2-like), c185243.graph_c0 (SWEET4), c167651.graph_c0 (SUC2-like), c174345.graph_c0 (SWEET1a). T These findings suggest that these genes play crucial roles in sucrose synthesis and accumulation during fruit development in *C. drupifera*. A study on sugar accumulation during developmental stages of *C. oleifera* plants also found significant enrichment of genes encoding SuSy enzymes (He et al., 2022), indicating that SuSy enzymes may be key regulators of sugar accumulation in Camellia species.

**Table 3:** The correlation analysis between gene expression levels and sucrose content.

Gene name	Correlation with sucrose content
Sugar Transporter Genes	
c159295.graph_c0 (SUC2-like)	0.94
c156402.graph_c0 (SUC2-like)	0.93
c185243.graph_c0 (SWEET4)	0.86
c167651.graph_c0 (SUC2-like)	0.74
c174345.graph_c0(SWEET1a)	0.6
Sucrose synthesis genes	
c158337.graph_c0 (SPS)	0.94
c166323.graph_c0 (SuSy),	0.91
c193943.graph_c0 (SPS)	0.88
c173428.graph_c0 (SuSy)	0.87
c192474.graph_c0 (SPS)	0.85

## 4 Discussion

During plant growth and development, photosynthates, particularly sugars, play a crucial role in the transport and accumulation among different organs, especially in oil crops, exerting a critical influence on lipid synthesis and conversion. However, as an important woody oilseed crop in southern China, systematic studies on the transport and accumulation of photosynthates in *C. drupifera*. during its growth stages and lipid synthesis phase are lacking. This study investigates the characteristics of photosynthate transport and accumulation in *C. drupifera*. during fruit growth and lipid synthesis phases, integrating organ-specific sugar content, activities of sugar metabolism-related enzymes, and transcriptomic data to elucidate key periods and regulatory mechanisms of lipid conversion in *C. drupifera*.

### 4.1 Characteristics of photosynthetic product transport and allocation

From 0 to 6 hours after ^13^C labeling, the leaves, serving as the source organ, synthesize a substantial amount of photosynthates and rapidly transport them to various sink organs. The *δ* ^13^C content in branches and seeds shows a rapid increase. Subsequently, the efficiency of transport decreases, with the critical transport period occurring within 48 hours of photosynthate synthesis by the leaves. The *δ* ^13^C in source leaves sharply within 24 hours by 90.23%, followed by a slower decline. The maximum *δ* ^13^C values in branches, fruit peels, and seeds are reached at 24 hours, 48 hours, and 48 hours after labeling, respectively. By this time, the ^13^C-labeled photosynthates synthesized in the source leaves have been mostly transported to various sink organs. After 48 hours, *δ* ^13^C decreases in all tissues due to the plant’s own respiratory and metabolic activities. Similar findings were reported in the study of *C. oleifer*a (He et al., 2022).

After 72 hours of labeling in July and August, the δ^13^C c content in leaves is significantly higher than in other organs. This disparity may be attributed to significant differences in source-sink activities during this period (Hidaka et al., 2019). In July and August, Camellia oleifera undergoes summer shoot growth, where leaves exhibit high demand for photosynthates, thereby displaying stronger sink activities. Photosynthates fixed and absorbed are predominantly utilized for their own growth. Similar observations were reported in the study of *Pinus massoniana* Lamb (Deng et al., 2020), where early allocation of photosynthates from photosynthesis was notably higher in leaves compared to other organs. In September and October, the δ^13^C c content in all organs turns negative, possibly due to decreased photosynthetic capacity of the source organs during these months, resulting in less fixation of ^13^CO2 provided artificially. The δ^13^C in plant tissues is also depleted due to respiratory and metabolic activities. Currently, the δ^13^C content in each organ is approximately at natural background levels.

The source-sink relationship hypothesis posits that the distribution of photosynthetic products is influenced by the supply capacity of sources, the competitive ability of sinks, and the transport capacity of phloem (Smith et al., 2018). Efficient allocation and transport of photosynthates to fruits are crucial for maximizing the yield potential of economic crops. Typically, sink strength is strongest in the growing centers of trees, where assimilates from leaves are preferentially allocated to support growth. In *Malus pumila* Mill, assimilates allocated from leaves to fruits initially increase and then decrease as fruits develop (Sha et al., 2021). Similarly, in *C. oleifer*a, the most intense competition for assimilates among organs occurs during the lipid conversion phase (Wen et al., 2021). The source-sink relationship affects the transport and distribution of assimilates. The proximity of leaves to fruits has a more significant impact on assimilate accumulation in fruits compared to pruning, with leaves closer to fruits contributing more to dry matter accumulation in fruits (Sha et al., 2021). In *C. oleifer*a, retaining source leaves and removing symmetrical branches improves single fruit weight and dimensions (Yuan et al., 2015). In this study, the center of photosynthate distribution in *C. drupifera* changes with fruit growth and lipid conversion processes. In the early stages of development, leaves exhibit stronger sink activity, retaining more assimilates to meet their growth and development needs. However, as fruits grow and demand for lipid synthesis increases, the growth center shifts towards seeds, leading to increased transport and accumulation of assimilates in seeds.

In *C. drupifera*, initially, a disproportionate amount of ^13^C assimilates flows towards the leaves, fulfilling the nutritional needs for plant growth. However, this allocation pattern can hinder nutrient transport towards seeds, potentially resulting in lower yield of *C. drupifera*. To mitigate this issue and enhance nutrient transport towards seeds for increased oil production, redistributing carbon among organs through strategic pruning is advantageous. This approach aims to optimize metabolic activities in sinks, improve the source-sink relationships, and ultimately enhance the productivity and economic efficiency of *C. drupifera*.

### 4.2 The relationship between sugar metabolism enzyme activity and sugar content.

The primary products of photosynthesis are carbohydrates, predominantly starch and soluble sugars such as glucose, sucrose, and fructose (Wen et al., 2021; Li et al., 2023). During the processes of fruit growth and oil synthesis in *C. drupifera*, significant variations are observed in the accumulation and metabolism of carbohydrates across different organs.

Soluble sugars represent the predominant form of carbohydrates in various organs of *C. drupifera* at different developmental stages (**Figures 4E, F**). In fruit peel and leaves, there is an initial increase in soluble sugar content followed by a subsequent decrease and then another increase. Conversely, in seeds, soluble sugars initially increase and subsequently show a slight decrease. This pattern likely reflects the prioritized utilization of sugars during oil synthesis: in seeds, soluble sugars are preferentially utilized for oil production. When soluble sugars become limited, starch is then converted to meet the demands of oil synthesis. Consequently, this leads to alternating accumulation phases of soluble sugars and starch in different organs (Eveland and Jackson, 2012; Guo et al., 2023).

At various developmental stages, *C. drupifera* exhibits distinct patterns of sugar accumulation among its organs. Typically, the highest concentration of soluble sugars is observed in the fruit peel, followed by seeds and leaves. Conversely, starch content tends to be highest in leaves, followed by seeds and fruit peel. This contrasting pattern of sugar accumulation in *C. drupifera* differs significantly from that observed in *C. oleifera* (He et al., 2022). These differences likely stem from specific variations in growth dynamics, developmental stages, and the timing of oil conversion processes between the two species. During the oil conversion phase, there is a notable accumulation of soluble sugars in the fruit of *C. drupifera*, particularly surpassing levels observed in its leaves (**Figures 4C, D**). This heightened accumulation is believed to facilitate enhanced cell division and expansion within the fruit. Consequently, these factors contribute to the larger physical dimensions observed in the fruits of *C. oleifera* compared to *C. drupifera*.

Soluble sugars, including glucose, sucrose, and fructose, play crucial roles in plant metabolism (Si et al., 2023; Yu et al., 2023). In *C oleifera*, the oil content shows a positive correlation with sucrose and starch levels, while it correlates negatively with reducing sugars like glucose (Song et al., 2021). During July and August, *C. drupifera* exhibits elevated levels of fructose and glucose but lower levels of sucrose. These reducing sugars serve as energy sources that fuel rapid fruit growth during this developmental stage. As development progresses, there is a notable increase in sucrose content within the seeds, providing essential energy and carbon precursors for oil synthesis. The high concentration of soluble sugars in the fruit peel may be attributed to its lignification process. As the fruit matures, the fruit peel undergoes further lignification, requiring increased glucose for cellulose and lignin synthesis. Concurrently, nutrient competition between the fruit peel and seeds enhances the translocation of sucrose to the fruit peel, thereby supporting its thickening process.

Sugar metabolism-related enzymes play pivotal roles in regulating sugar content across various plant organs (Jammer et al., 2020; Zhang et al., 2021; He et al., 2022; Ren et al., 2023). For instance, changes in the activities of sucrose synthase (SuSy) and sucrose phosphate synthase (SPS) are closely linked to sugar accumulation dynamics. In fruits of *Pyrus communis* L. and *C. oleifera*, increased SuSy activity has been observed to enhance sucrose accumulation (Lu et al., 2020; He et al., 2022), while in *M. pumila* Mill, elevated sucrose levels correlate with heightened SPS activity (Li et al., 2012). Studies on *C. oleifera* indicate that sucrose transport during early and late fruit development predominantly occurs through symplastic pathways, transitioning to apoplastic pathways in mid-stages (Si et al., 2023).

During late stages of fruit maturation, enhanced SuSy and SPS activities facilitate sucrose metabolism and conversion, thereby promoting lipid synthesis (He et al., 2022). Soluble acid invertase (S-AI) activity shows positive correlations with sucrose and glucose levels, contributing to the establishment of concentration gradients between source leaves and sink organs. In fruits, S-AI rapidly hydrolyzes unloaded sucrose into glucose and fructose (Liu et al., 2019). Cell-wall binding acid invertase (B-AI), primarily active in the phloem, is closely associated with apoplastic unloading processes (Wang et al., 2018).

In the late stages of *C. oleifera* fruit development, there is a notable increase in S-AI and NI activities. These enzymes promote vacuole expansion and sugar accumulation, crucial for fruit maturation and lipid deposition (Wang et al., 2018). Under conditions of low S-AI activity, NI becomes pivotal in sucrose breakdown (Winter and Huber, 2000; Zhang et al., 2019). In *C. drupifera* S-AI activity remains consistently low in leaves. Conversely, in fruit peel, although S-AI activity exhibits a declining trend, it maintains relatively high levels, resulting in lower sucrose and higher fructose and glucose contents in the peel. In seeds, S-AI activity increases steadily during initial development stages but decreases during lipid conversion, suggesting its role in cell expansion and seed maturation. NI activity in *C. drupifera* leaves is notably 5-10 times higher than S-AI activity, highlighting NI’s significant role in sucrose breakdown. NI activity remains consistently high in sink organs, facilitating sucrose concentration gradients in sieve tube-companion cell complexes, crucial for sucrose unloading (Hidaka et al., 2019; Wang et al., 2018). NI activity peaks in seeds in October, aiding sucrose transport and lipid formation. Notably, no NI activity was detected in fruit peel, possibly due to its immature tissue nature. In August and October, there is a significant rise in B-AI activity in *C. drupifera* seeds, promoting sucrose hydrolysis and facilitating sucrose transport from leaves to seeds. Consequently, soluble sugar content in seeds peaks in August, shifting towards lipid formation during the lipid conversion period. SuSy-II and SPS play diverse roles across different organs at various developmental stages (Mirajkar et al., 2016). SuSy-II shows a significant positive correlation with glucose content in leaves, sucrose content in fruit peel, and sucrose content in seeds, but a negative correlation with fructose and glucose contents. SPS mirrors SuSy-II in seeds but demonstrates an opposite role in leaves and fruit peel.

SuSy-II, SPS, and S-AI in *C. drupifera* demonstrate a significant positive correlation with sucrose content in seeds, as indicated in the **Table 3**. Notably, SuSy-II activity remains consistently high throughout the development stages. This persistent activity positively correlates with sucrose content not only in seeds but also in fruit peel, underscoring its pivotal role in sucrose accumulation within fruits. This observation aligns with findings from studies on sugar beet roots, where similar relationships between sugar metabolism enzyme activity and sugar content have been reported (Shao et al., 2020). Moreover, previous research has highlighted that oil content in *C. oleifera* fruits shows positive associations with sucrose and starch content, while negatively correlating with reducing sugar content (Song et al., 2021). These findings suggest that SuSy enzymes influence lipid accumulation in seeds by modulating sucrose levels in fruits.

During the lipid conversion period of *C. drupifera* seeds, sucrose content stands notably higher compared to other sugars, whereas the fruit peel shows an opposite trend with lower sucrose and higher soluble sugar levels. This disparity forms the basis for nutrient dynamics during seed development, influencing the activities of SuSy-II and SPS enzymes crucial for lipid synthesis. The fruit peel’s low sucrose content and elevated soluble sugars promote lignification and nutrient competition, thereby contributing to the thickened peel and extended fruit morphology observed in *C. drupifera*. This phenomenon helps explain the development of large fruits with robust peels in this species.

### 4.3 Analysis of the expression of sugar metabolism enzymes and sugar transport protein gene families during the development and maturation process of *C. drupifera* fruits

Sucrose serves dual roles in plants as both a metabolite and a signaling molecule, capable of initiating signaling pathways that trigger changes in gene expression and physiological adaptations (Wind et al., 2010). In the study of *C. drupifera*, gene expression analysis conducted throughout fruit growth, development, and oil conversion aims to pinpoint critical periods and the relevant genes responsible for regulating sugar content and transport (**Figure 11**). Genes encoding sucrose synthase (SuSy), sucrose phosphate synthase (SPS), and sugar transport proteins exhibit heightened expression during the oil conversion stage of fruit development. The peak sucrose content observed in seeds during September suggests this month is pivotal for sucrose accumulation in fruits (**Figure 5A**), facilitating the completion of oil conversion. Furthermore, analysis of gene expression profiles reveals a notable enrichment of genes involved in signal transduction mechanisms (**Figure 8**), underscoring sucrose’s role as a likely signaling molecule initiating metabolic activities. Throughout later developmental stages sustained high levels of sucrose content, enzyme activities related to sugar metabolism, and the expression of their corresponding genes indicate ongoing accumulation and transport of sucrose into *C. drupifera* seeds.

Enzymes such as INV, SuSy, SPS, SPP, HXK, and FRK play crucial roles in regulating sucrose levels through synthesis (positive regulation) or degradation (negative regulation) pathways (Ren et al., 2022; Zhang et al., 2023). INV enzymes (NI, S-AI, B-AI) irreversibly hydrolyze sucrose into glucose and fructose, whereas SuSy enzymes reversibly cleave sucrose into UDP-glucose and fructose (Li et al., 2012; He et al., 2022). High expression of SuSy has been linked to increased sucrose accumulation in *P. sorotina* and *C. oleifera* fruits (Lu et al., 2020; He et al., 2022), while in *M. pumila*, elevated SPS expression correlates with rapid sucrose accumulation (Li et al., 2012). Research on *C. oleifera* has demonstrated sucrose’s role as a signaling molecule initiating sugar metabolism processes. SuSy-II and SPS enzymes are pivotal in sucrose synthesis and accumulation, with heightened expression typically observed during late stages of *C oleifera* fruit development (He et al., 2022). In the context of *C. drupifera* seed development, correlation analysis between sucrose content and gene expression levels has identified significant positive correlations (**Table 3**) with specific sugar metabolism enzyme genes: c158337.graph_c0 (SPS), c166323.graph_c0 (SuSy), c193943.graph_c0 (SPS), c173428.graph_c0 (SuSy), c192474.graph_c0 (SPS). Among these, c158337.graph_c0 (SPS) (r^2^ = 0.94 with sucrose content) and c166323.graph_c0 (SuSy) (r^2^ = 0.91 with sucrose content) a are presumed to be pivotal in sucrose synthesis and accumulation during *C. drupifera* fruit development. Studies on sugar accumulation in *C. oleifera* have similarly highlighted significant enrichment of SuSy enzyme genes (He et al., 2022)., suggesting their crucial regulatory role in sucrose accumulation in *camellia* plants.

In *Arabidopsis thaliana* (L.) Heynh, sucrose transport from parenchyma cells of the phloem to the apoplast/cell wall is facilitated by SWEETs, followed by absorption into sink organs via sucrose transporters (SUTs/SUCs), thereby distributing sucrose to various tissues (Singh et al., 2023). Specifically, SUT1, a member of the SUT1 subfamily known for its high affinity for sucrose in dicotyledonous plants, and SUC2 are crucial for this process, with SWEET11 and SWEET12 facilitating sucrose entry into the apoplast before transport into companion cells (Chen et al., 2022; Ye et al., 2023). These transporters are pivotal for sugar accumulation in various organs of plants like *C. oleifera* flower buds (Wu et al., 2022).

In *C. drupifera*, a gene expression heatmap of fruit development reveals significant variations in sucrose transporter expression across different developmental stages (**Figure 11**). Notably, genes c159295.graph_c0 (SUC2-like), c156402.graph_c0 (SUC2-like), c185243.graph_c0 (SWEET4), c167651.graph_c0 (SUC2-like), and c174345.graph_c0 (SWEET1a) show strong correlations with sucrose content in seeds (**Table 3**). Among these, c159295.graph_c0 (SUC2-like) (r^2^ = 0.94 with sucrose content), c156402.graph_c0 (SUC2-like) (r^2^ = 0.93 with sucrose content) exhibit peak expression during the oil synthesis phase, suggesting their pivotal role in maintaining dynamic sucrose balance in *C. drupifera* seeds. These genes are identified as critical regulators involved in sucrose transport mechanisms and maintaining optimal sucrose levels in fruits.

**Figure 12:**
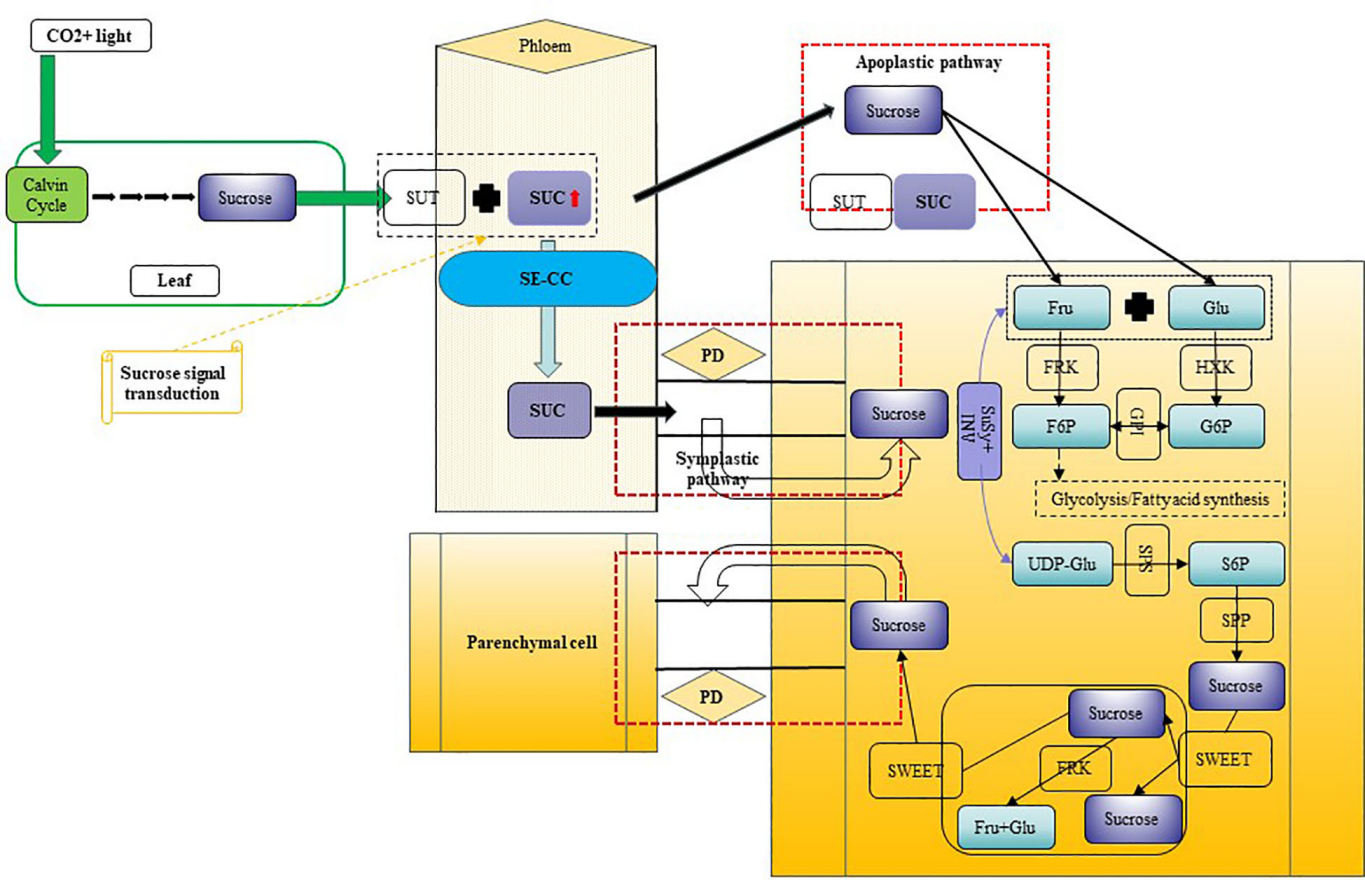
Hypothetical model of sucrose synthesis, transport, and metabolism in *C. drupifera* fruit development. Synthesis of sucrose in source organs simultaneously triggers signal transduction, leading to the expression of SUTs. Sucrose is loaded into the phloem via both symplastic and apoplastic pathways, and subsequently enters the cells of sink organs. SuSy and INV enzymes jointly regulate the variation in sucrose content during different developmental stages. Main Components and Processes in This Model: Sucrose, Sucrose; SWEET, SUT, SUC, Sucrose transport proteins (with SUC being a critical sucrose transporter); SE/CC, Sieve Element/Companion Cell; PD, Plasmodesmata; B-AI, Cell Wall Acid Invertase; Fru, Fructose; Glu, Glucose; F6P, Fructose-6-phosphate; G6P, Glucose-6-phosphate; S6P, Sucrose-6-phosphate; FRK, Fructokinase; HXK, Hexokinase; GPI, Glucose-6-phosphate Isomerase; SuSy, Sucrose Synthase; SPS, Sucrose Phosphate Synthase; SPP, Sucrose Phosphatase; UDP-Glu, Uridine-5’-diphosphate-glucose.

Based on our research on *Camellia oleifera*, research group has proposed a model for sucrose transport and metabolism in fruits, illustrated in **Figure 12**. Sucrose transport in fruits occurs through two primary pathways: symplastic and apoplastic. The symplastic pathway utilizes plasmodesmata (PD) for sucrose movement, whereas the apoplastic pathway relies on SUC sucrose transporters. Within cells, intracellular sucrose levels are predominantly regulated by enzymes such as SuSy and SPS. This study establishes a critical genetic and enzymatic framework for comprehending sugar metabolism and accumulation during the growth, development, and oil conversion phases in *C. drupifera* fruits.

## 5 Conclusion

The leaves of *C. drupifera* efficiently assimilate photosynthetic products within 0 to 6 hours, with peak transport rates during this period gradually declining thereafter, notably dropping after 48 hours. Various organs at different developmental stages of *C. drupifera* exhibit significant disparities in the accumulation of photosynthetic products. During the rapid growth phase of fruits, leaves serve as the primary site for accumulation, whereas during the oil conversion phase, seeds become the predominant storage organ. Soluble sugar content generally surpasses starch content across all organs, with fruits and leaves accumulating primarily fructose and glucose, while seeds predominantly store sucrose. Initially, seeds accumulate fructose and glucose, transitioning to sucrose accumulation in later stages. The expression of SuSy-II, SPS, and S-AI genes positively correlates with sucrose content in seeds, notably SuSy-II showing significant correlations with sucrose levels in both seeds and fruit peels. Moreover, through the association between sucrose content and gene expression, we have identified 10 crucial genes (DEGs) involved in sugar metabolism in *C. drupifera*, Notably, c158337.graph_c0 (SPS) (r^2^ = 0.94 with sucrose content), c166323.graph_c0 (SuSy) (r^2^ = 0.91 with sucrose content), c159295.graph_c0 (SUC2-like) (r^2^ = 0.94 with sucrose content), c156402.graph_c0 (SUC2-like) (r^2^ = 0.93 with sucrose content) exhibit heightened expression during the oil accumulation phase, crucial for maintaining dynamic sucrose balance in seeds. These genes are identified as key regulators governing sucrose transport in *C. drupifera* and ensuring sucrose content stability in fruits.

However, despite comprehensive evaluations of plant gene expression related to sugar accumulation in *C. drupifera* through physiological and transcriptomic analyses, the intricate regulatory networks governing sugar accumulation and oil synthesis in plants warrant further exploration. A deeper understanding of the interconnections among these genes and their functional roles is essential for elucidating the comprehensive mechanisms involved.

## Data Availability

The datasets presented in this study can be found in online repositories. The names of the repository/repositories and accession number(s) can be found in the article/supplementary material.
